# Quality assessment on Polygoni Multiflori Caulis using HPLC/UV/MS combined with principle component analysis

**DOI:** 10.1186/1752-153X-7-106

**Published:** 2013-06-24

**Authors:** Yang Zhao, Chun-Pin Kao, Yuan-Shiun Chang, Yu-Ling Ho

**Affiliations:** 1 Department of Chinese Pharmaceutical Sciences and Chinese Medicine Resources, College of Pharmacy, China Medical University, Taichung 40402, Taiwan; 2 Department of Nursing, Hsin Sheng College of Medical Care and Management, Taoyuan 32544, Taiwan; 3 Chinese Crude Drug Pharmacy, China Medical University Hospital, Taichung 40402, Taiwan; 4 Department of Nursing, Hungkuang University, Taichung 43302, Taiwan

**Keywords:** Polygoni Multiflori Caulis, Quality assessment, HPLC/UV/MS, Principle component analysis

## Abstract

**Background:**

Polygoni Multiflori Caulis, the dried caulis of *Polygonum multiflorum* Thunb., is one of the commonly used traditional Chinese medicines having antioxidant, anti-obesity, anti-inflammatory and antibacterial effects. Polygoni Multiflori Caulis used clinically or circulated on market have great differences in their diameters. However, to the best of our knowledge, no study has been reported on the qualities of Polygoni Multiflori Caulis with different diameters.

**Results:**

Systematic HPLC/UV/MS chromatographic fingerprinting and quantitative analytical methods combined with principal component analysis were developed and applied to analyze different Polygoni Multiflori Caulis samples. The contents of 2,3,5,4′-tetrahydroxystilbene-2-O-*β*-D-glucoside, the chemical marker for quality control on Polygoni Multiflori Caulis specified in Chinese Pharmacopoeia (2010 edition), were found to have surprising relevance with the samples’ diameters for the first time.

**Conclusion:**

The finding provides a scientific basis for collecting Polygoni Multiflori Caulis in the best time. Moreover, the diameter can be used as the criterion for quality control on Polygoni Multiflori Caulis as a preliminary step in the future. In addition, scores plot obtained from principal component analysis shows the obvious differences between unqualified Polygoni Multiflori Caulis samples and qualified ones visually, which can be used to single out the unqualified ones with qualified ones efficiently and immediately.

## Background

Polygoni Multiflori Caulis (PMC), *Shou*-*Wu*-*Teng* in Chinese, is the dried caulis of *Polygonum multiflorum* Thunb. It is one of the commonly used traditional Chinese medicines (TCMs) listed in Chinese Pharmacopoeia (CP) (2010 edition) [1]. Pharmacological studies indicated that it had antioxidant [2,3], anti-obesity [4], anti-inflammatory and antibacterial effects [5].

Anthraquinones, flavonoids and stilbene glycosides are considered to be the main active constituents in PMC [6,7]. However, unlike Polygoni Multiflori Radix, *He*-*Shou*-*Wu* in Chinese, there are only a few reports on quality control of PMC. High performance liquid chromatography (HPLC) with ultraviolet detector (UV) were applied to determine the contents of 2,3,5,4′-tetrahydroxystilbene-2-O-*β*-D-glucoside (THSG) and emodin [8-10], but THSG, one of the stilbene glycoside, was not specified as chemical marker for quantitative determination of PMC until CP (2010 edition) was published.

As for original plant morphology, it is regulated in CP (2010 edition) that the diameter of PMC is between 4 and 7 mm. However, it derives from *P*. *multiflorum* which is a perennial plant and can be harvested all the year round, so PMC circulated on market have big variations on their diameters. In the PMC samples we collected, the smallest diameter is just 0.5 mm, however, the biggest one reaches to 36 mm. Their differences go so far to 70 times unexpectedly. In that way, are there any differences on their qualities? The issue arouses our great interest.

In the present study, chromatographic fingerprinting and quantitative analytical methods were developed to analyze different PMC samples. Seven peaks, marked as 1 to 7, were designated as characteristic peaks in chromatographic fingerprints. They were identified as THSG, emodin-8-O-*β*-D-glucoside, emodin-8-O-(6′-O-malonyl)-*β*-D-glucoside, physcion-8-O-*β*-D-glucoside, physcion-8-O-(6′-O-acetyl)-*β*-D-glucoside, emodin and physcion, respectively, based on UV and MS data compared with reference compounds and related literatures [11-17]. THSG, emodin and physcion were quantified at their maximal UV wavelengths. From the results, we found that the contents of THSG had great relevance with the diameters of PMC samples. Principal component analysis (PCA), one of the popular chemometrics, was then used for comprehensive and systematic assessment on PMC samples collected from different regions with different diameters, based on the variables including the contents of the three quantified analytes and the PA/W (peak area divided by sample weight) values of the four unquantified ones. Very useful information were obtained from PCA scores plot, by which unqualified PMC samples could be distinguished from qualified ones visually and immediately. Points of view how variables contributed to samples’ positions in scores plot were also discussed in detail according to PCA loadings plot.

## Experimental

### Chemicals, solvents and herbal materials

THSG, emodin and physcion were purchased from Shanghai R & D Center for Standardization of Traditional Chinese Medicines. LC-grade methanol, acetonitrile, formic acid and phosphoric acid were purchased from the branch company of Merck in Taipei, Taiwan. Purified water was prepared with Milli-Q system (Millipore, Milford, MA, USA). All other reagents used in the present study were of analytical grade. Herbal materials of PMC were collected from different regions of mainland China and local pharmacies of Hong Kong, which were marked as PMC-01 to PMC-08 and L-PMC-01 to L-PMC-11, respectively. The detailed information of the samples is summarized in Table 1. All the plant specimens have been deposited in Department of Chinese Pharmaceutical Sciences and Chinese Medicine Resources, School of Pharmacy, China Medical University.

**Table 1:** Collected information of the nineteen PMC samples and the content (%) of THSG, emodin and physcion in the samples

**Sample No.**	**Content (mg/g dry weight) (Mean ± SD)**			**Origin**	**Type**	**Diameter (mm)**	**Moisture content (%)**
	**THSG**	**Emodin**	**Physcion**				
PMC-01	19.547 ± 0.0016	0.791 ± 0.0003	0.785 ± 0.0001	Zhejiang	Raw Material ^a^	3 ~ 9	11.10
PMC-02	23.766 ± 0.0116	1.295 ± 0.0007	1.394 ± 0.0005	Yunnan	Raw Material	3 ~ 9	10.18
PMC-03	0.742 ± 0.0007	0.023 ± 0.0001	ND	Guangxi	Medicinal Slices ^b^	7 ~ 12	8.23
PMC-04	1.275 ± 0.0007	0.054 ± 0.0001	0.043 ± 0.0000	Sichuan	Raw Material	0.5 ~ 2	9.32
PMC-05	0.351 ± 0.0004	0.015 ± 0.0000	ND	Sichuan	Raw Material	3 ~ 9	9.98
PMC-06	0.268 ± 0.0001	0.074 ± 0.0000	0.086 ± 0.0000	Jiangsu	Medicinal Slices	3 ~ 11	11.66
PMC-07	5.955 ± 0.0022	0.452 ± 0.0002	0.592 ± 0.0005	Sichuan	Raw Material	4 ~ 8	10.09
PMC-08	11.184 ± 0.0070	0.424 ± 0.0004	0.836 ± 0.0008	Hu’nan	Raw Material	2 ~ 5	8.06
L-PMC-01	1.195 ± 0.0032	0.031 ± 0.0000	0.102 ± 0.0002	Guangxi	Medicinal Slices	11 ~ 26	7.66
L-PMC-02	0.427 ± 0.0003	0.010 ± 0.0000	ND	Guangdong	Medicinal Slices	9 ~ 27	8.03
L-PMC-03	0.501 ± 0.0002	0.025 ± 0.0001	0.015 ± 0.0001	Yunnan	Medicinal Slices	10 ~ 26	8.20
L-PMC-04	1.482 ± 0.0011	0.039 ± 0.0000	0.042 ± 0.0000	Guangxi	Raw Material	4 ~ 14	7.94
L-PMC-05	0.880 ± 0.0005	0.027 ± 0.0000	0.013 ± 0.0000	He’nan	Medicinal Slices	10 ~ 28	7.64
L-PMC-06	1.683 ± 0.0014	0.014 ± 0.0000	ND	Unknown	Medicinal Slices	12 ~ 26	9.25
L-PMC-07	ND	ND	ND	Guangxi	Raw Material	7 ~ 36	8.67
L-PMC-08	0.840 ± 0.0005	ND	ND	Hu’nan	Medicinal Slices	4 ~ 14	8.87
L-PMC-09	ND	ND	ND	Guangxi	Raw Material	8 ~ 23	8.53
L-PMC-10	ND	ND	ND	Unknown	Raw Material	7 ~ 15	7.62
L-PMC-11	41.361 ± 0.0009	0.864 ± 0.0009	1.054 ± 0.0006	Unknown	Raw Material	2 ~ 6	8.74

^a^ Tied into a bundle. Cut into small pieces and dried in shade places before experiments.

^b^ Purchased from markets or pharmacies.

### Sample preparation

Dried PMC samples were sliced into small pieces and were ground into fine powders (20 mesh) using a grinder with a knife blade. Half gram of each PMC powder was carefully weighed into a 50 mL centrifuge tube. Twenty microliters of 75% methanol was then added into the tube and shaken briefly to mix. Each sample was then sonicated in an ultrasonic cleaner (Delta DC400H) at a frequency of 40 kHz at 25°C for 30 min. The extract was centrifuged for 10 min at 3000 rpm and the supernatant was then transferred into a 50 mL volumetric flask. The procedure was repeated for one more time and the supernatants were combined. The final volume was made up to 50 mL with 75% methanol. The final combined extract was filtered through a 0.45 μm PVDF syringer filter (VWR Scientific, Seattle, WA) before analysis. An aliquot of 10 μL solution of each sample was used for HPLC and HPLC-ESI-MS analyses.

### Standards solutions

Stock standard solutions of the three accurately weighed reference compounds were prepared in 75% methanol. A standard mixture was obtained by mixing the individual stock standard solution to give THSG at a concentration of 252.5 mg/L, emodin at 138.325 mg/L and physcion at 15.4 mg/L. The standard mixture was diluted with 75% methanol to appropriate concentrations for calibration curves. The solutions were brought to room temperature and filtered through 0.45 μm PVDF syringer filter and an aliquot of 10 μL of each solution was used for HPLC analysis.

### HPLC analysis

HPLC analyses were performed on a Waters 2695 HPLC system equipped with Waters 2998 photodiode array detector (PDA), Waters e2695 separations module and column heater module. A Grace Alltima C18 column (250 mm × 4.6 mm i.d., 5 μm) was used. The mobile phase consisted of 0.5% *v*/*v* formic acid aqueous solution (A) and acetonitrile (B). The optimized elution conditions were as follow: 0–22 min, 16% B; 22–45 min, 16-34% B; 45–60 min, 34-38% B; 60–70 min, 38-95%; 70–80 min, 95% B. The flow rate was 1 mL/min and the injection volume was 10 μL. UV spectra were acquired from 190 nm to 400 nm. The autosampler and column compartment were maintained at 25°C and 35°C, respectively.

### HPLC-ESI-MS analysis

HPLC-ESI-MS analyses were performed on a TSQ Quantum Access Max Triple Stage Quadrupole Mass Spectrometer (Thermo Fisher Scientific Inc., Waltham, MA, USA) with an Accela 1250 UHPLC system equipped with an Accela 1250 photo diode array (PDA) detector, an Accela HTC PAL autosampler, and an Accela 1250 binary pump. The column and elution conditions used were the same as those used in “HPLC analysis” except that the flow rate was set at 0.25 mL/min with a split ratio. Ultrahigh pure helium (He) and high purity nitrogen (N_2_) were used as collision gas and for nebulizer, respectively. The optimized parameters in negative/positive ion modes were as follows: ion spray voltage, -2.5 kV/3.0 kV; auxiliary gas, 40 arbitrary units; sheath gas, 15 arbitrary units; capillary temperature, 350°C; vaporizer temperature, 350°C; capillary offset, -35 V/18 V; tube lens offset, -33 V/102 V. Spectra were recorded in the range of *m*/*z* 100–1000 for full scan data, meanwhile, the normalized collision energy was set at 45% for MS^2^ data with dependant scan.

### Quantitative analytical method validation

The limits of detection (LOD) and quantitation (LOQ) were defined as the lowest concentrations of analytes in the sample that can be detected and quantified, which were determined on the basis of signal-to-noise ratios (S/N) at 3:1 and 10:1, respectively. Intra- and inter-day variations were chosen to evaluate the precision of the developed method. The intra-day variation was determined by analyzing one of the mixed stock solutions (THSG at 50.5 mg/L, emodin at 27.665 mg/L and physcion at 3.08 mg/L) five times within one day. While for inter-day variability test, the same solution was examined in triplicate for three consecutive days. Repeatability was confirmed with five different working solutions prepared from sample PMC-01. Stability was tested with the same sample solution at 0, 2, 4, 8, 12, 24 h.

### Fingerprinting and principal component analyses

The data obtained from chromatographic fingerprints were analyzed with Solo (Eigenvector Research, Inc.,Wenatchee, WA) for chemometric analysis. Normalize (2-Norm, length = 1) and mean center were used for data reprocessing before principal component analysis (PCA) was performed.

## Results and discussion

### Optimization of extraction method

The extraction solvents were optimized based on the extraction efficiency of THSG. Four solvents, ethanol, 50% methanol, 75% methanol and methanol were investigated with sonication at room temperature for 30 min. As a result, 75% methanol was proved to be superior to other solvents (Additional file 1: Figure S1 A and Figure S1 B), which was selected as the extraction solvent. The optimal extraction times for 75% methanol was further investigated. The powder of Polygoni Multiflori Caulis (0.5 g) was extracted with 20 mL of 75% methanol for three times (30 min for each time). It showed that most THSG was extracted (> 99%) after the second extraction. (Additional file 1: Figure S1 C). Finally, the optimal extraction method was finalized, as described in “Sample preparation”.

### Optimization of chromatographic conditions

**Figure 1:**
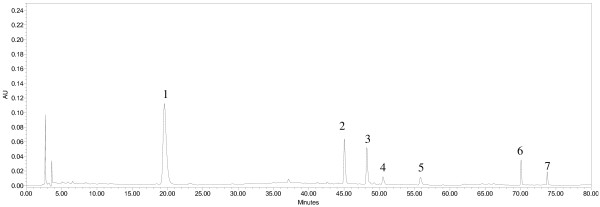
**Representative chromatographic fingerprint obtained from PMC-01 detected at 290 nm.** Seven characteristic peaks are marked as 1 to 7.

To develop a reliable chromatographic fingerprinting method, an optimized strategy for HPLC conditions was performed. To obtain sharp and symmetrical peaks, different mobile phase systems, including methanol–water, acetonitrile–water, acetonitrile–water (containing 0.5% formic acid, v/v) and acetonitrile–water (containing 0.1% phosphoric acid, v/v) were tested. As a result, high resolution, good baseline, sharp and symmetrical peaks were obtained by using acetonitrile–water (containing 0.5% formic acid, v/v) system. A few columns (Waters XBridge Shield RP18, Waters XTerra RP18, Thermo Ascentis C18) were screened before Grace Alltima C18 column (250 mm × 4.6 mm i.d., 5 μm) was finally selected as the column of choice. To obtain a sufficient large number of detectable peaks on the chromatographic fingerprints, PDA full scan (190–400 nm) was used for investigating all the main peaks and finally 290 nm was selected as detection wavelength. Representative chromatographic fingerprint obtained from PMC-01 is shown in Figure 1. Characteristic chemical compounds are marked as 1 to 7. In quantitative analysis, THSG was monitored at 320 nm, meanwhile, emodin and physcion were monitored at 290 nm. Different column temperatures at 20, 25, 30 and 35°C were also investigated. Although chromatograms detected at different temperatures didn’t show obvious differences, 35°C was selected as the preferable one in order to minimize the influences from room temperature on the chromatograms. In the process of gradient optimization, gradient time, gradient procedure and initial composition of the mobile phase were taken into consideration. Finally, the gradient procedure was finalized, as described in “HPLC Analysis”.

### Assignments of the seven characteristic peaks

Figure 1 shows the seven characteristic peaks detected at 290 nm in PMC-01. The structural identification of each peak was carried out by careful studies on MS and MS^2^ spectra and by comparison with reference compounds and literatures (Table 2). Under the optimized MS conditions, both negative and positive ESI modes were used in our experiment.

**Table 2:** Assignments of the seven characteristic peaks by HPLC/UV/MS

**No.**	**RT (min)**	**UV λ**_**max **_**(nm)**	**MS in Neg. mode**	**MS**^**2 **^**in Neg. mode**	**MS in Pos. mode**	**MS**^**2 **^**in Pos. mode**	**Assignment**	**References**
1	19.6	319	405 [M-H]^-^	243 [M-H-glucosyl]^-^	407 [M + H]^+^	245 [M + H-glucosyl]^+^	THSG	[11-14]
			451 [M-H + HCOOH]^-^		429 [M + Na]^+^			
			811 [2 M-H]^-^		245 [M + H-glucosyl]^+^			
2	45.1	281	431 [M-H]^-^	182 [M-H-glucosyl-CO_2_-CO-CH_3_]^-^	433 [M + H]^+^		Emodin-8-O-*β*-D-glucoside	[11,13-16]
				197 [M-H-glucosyl-CO_2_-CO]^-^	455 [M + Na]^+^			
				225 [M-H-glucosyl-CO_2_]^-^	887 [2 M + Na]^+^			
				241 [M-H-glucosyl-CO]^-^	271 [M + H-glucosyl]^+^			
				269 [M-H-glucosyl]^-^				
			863 [2 M-H]^-^					
3	48.2	281	517 [M-H]^-^	225 [M-H-malonylglucosyl-CO_2_]^-^	541 [M + Na]^+^		Emodin-8-O-(6′-O-malonyl)-*β*-D-glucoside	[14]
				269 [M-H-malonylglucosyl]^-^	271 [M + H-malonylglucosyl]^+^			
			473 [M-H-CO_2_]^-^					
4	50.5	270	445 [M-H]^-^	212 [M-H-glucosyl-CH_3_-2CO]^-^	469 [M + Na]^+^		Physcion-8-O-*β*-D-glucoside	[11,13-16]
				240 [M-H-glucosyl-CH_3_-CO]^-^	285 [M + H-glucosyl]^+^			
				253 [M-H-glucosyl-2CH_3_]^-^				
				268 [M-H-glucosyl-CH_3_]^-^				
				283 [M-H-glucosyl]^-^				
			491 [M-H + HCOOH]^-^					
			283 [M-H-glucosyl]^-^					
5	55.8	270	487 [M-H]^-^	240 [M-H-acetylglucosyl-CO-CH_3_]^-^	511 [M + Na]^+^		Physcion-8-O-(6′-O-acetyl)-*β*-D-glucoside	[11,16]
			533 [M-H + HCOOH]^-^		285 [M + H-acetylglucosyl]^+^			
			283 [M-H-acetylglucosyl]^-^					
6	70.1	222, 288	269 [M-H]^-^	182 [M-H-CO-CO_2_-CH_3_]^-^	271 [M + H]^+^		Emodin	[11-17]
				225 [M-H-CO_2_]^-^				
				241 [M-H-CO]^-^				
7	73.8	223, 286	283 [M-H]^-^	212 [M-H-2CO-CH_3_]^-^	285 [M + H]^+^		Physcion	[12-17]
				240 [M-H-CO-CH_3_]^-^	307 [M + Na]^+^			
				255 [M-H-CO]^-^				

Peak 1 occurs at retention time of 19.6 min with maximal UV absorption at 319 nm. In negative ion mode, the deprotonated molecular ion at *m*/*z* 405 [M-H]^-^, formic acid adduct ion at *m*/*z* 451 [M-H + HCOOH]^-^ and 811 [2 M-H]^-^ were found in its MS spectrum. Fragmentation of the ion at *m*/*z* 405 [M-H]^-^ yielded a product ion at *m*/*z* 243 arising from the loss of a glucosyl (−C_6_H_10_O_5_) unit. In positive ion mode, the protonated molecular ion at *m*/*z* 407 [M + H]^+^ and a sodium adduct ion at *m*/*z* 429 [M + Na]^+^ were found in its MS spectrum. The MS^2^ fragmentation of the ion at *m*/*z* 407 was further investigated and a dominant product ion at *m*/*z* 245 [M + H-glucosyl]^+^ was observed, corresponding to the loss of the glucosyl unit (162 amu). This peak was unequivocally identified as THSG by comparison with MS data of the standard as well as literatures [11-14].

**Figure 2:**
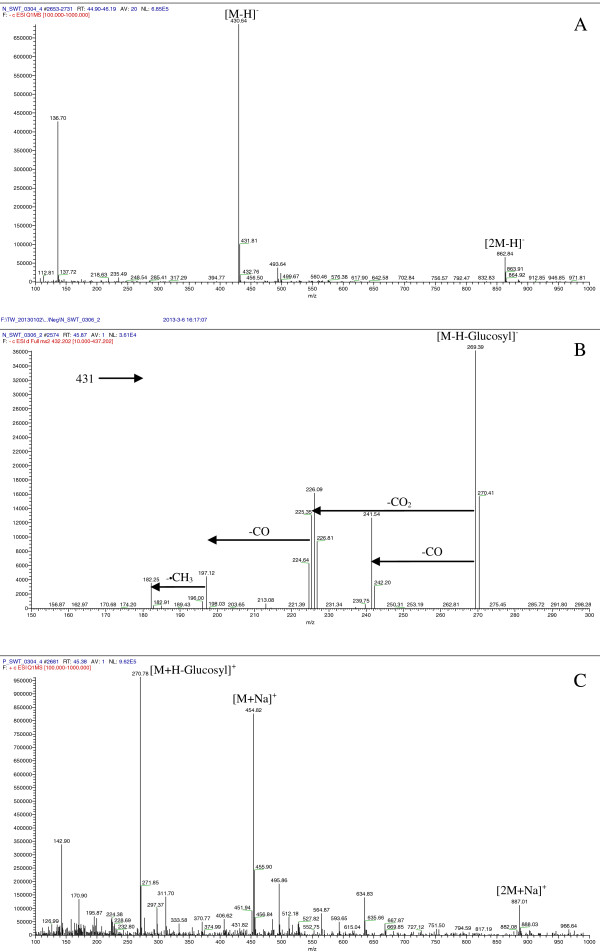
**Mass spectra of emodin-8-O-*β*-D-glucoside (peak 2) in (−)-ESI-MS (A), (−)-ESI-MS**^**2 **^**(B) and (+)-ESI-MS (C).**

Peak 2 shows the retention time of 45.1 min with maximal UV absorption at 281 nm. This peak gave [M-H]^-^ ion at *m*/*z* 431 and [2 M-H]^-^ ion at *m*/*z* 863 in MS spectrum in negative ion mode (Figure 2A). The ion at *m*/*z* 431 generated a series of fragment ions in its MS^2^ spectrum at *m*/*z* 269 [M-H-glucosyl]^-^, 241 [M-H-glucosyl-CO]^-^, 225 [M-H-glucosyl-CO_2_]^-^, 197 [M-H-glucosyl-CO_2_-CO]^-^ and 182 [M-H-glucosyl-CO_2_-CO-CH_3_]^-^ (Figure 2B). In positive ion mode, peak 2 produced a very week [M + H]^+^ ion but yielded prominent ions at *m*/*z* 455 [M + Na]^+^, 887 [2 M + Na]^+^ and 271 [M + H-glucosyl]^+^ in its MS spectrum (Figure 2C). Consequently, it was characterized as emodin-8-O-*β*-D-glucoside, of which some of the MS fragmentation behaviors were described in published papers [11,13-16].

Peak 3 occurs at retention time of 48.2 min with maximal UV absorption at 281 nm. Ions at *m*/*z* 517 and 473 were observed in its MS spectrum in negative ion mode, which were speculated as [M-H]^-^ and [M-H-CO_2_]^-^ ions, respectively. The ions at *m*/*z* 269 [M-H-malonylglucosyl]^-^ and 225 [M-H-malonylglucosyl-CO_2_]^-^ were found in its MS^2^ spectrum. Protonated molecular ion was not found in its MS spectrum in positive mode, but sodium adduct ion at *m*/*z* 541 [M + Na]^+^ and the one which lost a malonylglucosyl unit at *m*/*z* 271 [M + H-malonylglucosyl]^+^ were predominant. This peak was tentatively identified as emodin-8-O-(6′-O-malonyl)-*β*-D-glucoside based on its MS data and the literature [14].

**Figure 3:**
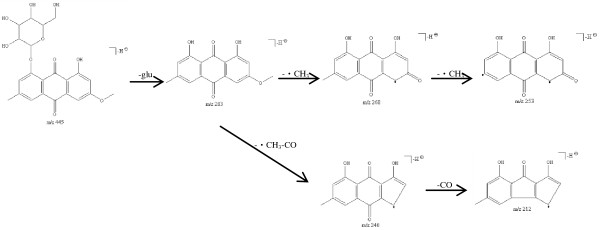
**The proposed fragmentation pathway for the deprotonated ion at *m/z *445 [M-H]**^-^**of physcion**-**8-O-*β-*D-glucoside.**

Peak 4 occurs at retention time of 50.5 min with maximal UV absorption at 270 nm. In negative ion ESI experiments, it yielded prominent deprotonated molecular ion at *m*/*z* 445 [M-H]^-^, formic acid adduct ion at *m*/*z* 491 [M-H + HCOOH]^-^ and the ion at *m*/*z* 283 [M-H-glucosyl]^-^. The MS^2^ spectrum of the ion at *m*/*z* 445 showed characteristic ions at *m*/*z* 283 [M-H-glucosyl]^-^, 268 [M-H-glucosyl-CH_3_]^-^, 253 [M-H-glucosyl-2CH_3_]^-^, 240 [M-H-glucosyl-CH_3_-CO]^-^ and 212 [M-H-glucosyl-CH_3_-2CO]^-^. In positive ion mode, the protonated molecular ion at *m*/*z* 447 was not found but the sodium adduct ion at *m*/*z* 469 [M + Na]^+^ and the ion at *m*/*z* 285 [M + H-glucosyl]^+^ were observed as predominant ions in MS spectrum. This peak was tentatively identified as physcion-8-O-*β*-D-glucoside based on the data mentioned above and the literatures [11,13-16]. A proposed fragmentation pathway for the deprotonated ion at *m*/*z* 445 [M-H]^-^ of physcion-8-O-*β*-D-glucoside is shown in Figure 3.

Peak 5 shows the retention time of 55.8 min with maximal UV absorption at 270 nm. Characteristic ions at *m*/*z* 487 [M-H]^-^, 533 [M-H + HCOOH]^-^ and 283 [M-H-acetylglucosyl]^-^ were produced from this peak in MS spectrum in negative ion mode. The deprotonated ion at *m*/*z* 487 [M-H]^-^ gave a predominant ion at *m*/*z* 240 in MS^2^ spectrum resulting from the losses of a acetylglucosyl unit, a neutral molecular of CO and a methyl group. In positive ion mode, we did not find the protonated molecular ion, however it yielded a predominant sodium adduct ion at *m*/*z* 511 [M + Na]^+^ and the ion at *m*/*z* 285 by losing a acetylglucosyl unit. By comparison with the reported paper [11,16], the peak was identified as physcion-8-O-(6′-O-acetyl)-*β*-D-glucoside.

Peak 6 was eluted at retention time of 70.1 min with maximal UV absorption at 222 and 288 nm, which produced the [M-H]^-^ ion at 269 in the MS spectrum in negative ion mode. It further gave fragment ions at *m*/*z* 241 [M-H-CO]^-^, 225 [M-H-CO_2_]^-^ and 182 [M-H-CO-CO_2_-CH_3_]^-^ in the MS^2^ spectrum. In positive ion mode, the peak yielded weak protonated molecular ion at *m*/*z* 271 [M + H]^+^ in MS spectrum, and no useful information was obtained in its MS^2^ spectrum. By comparison with MS behaviors of the standard and the literatures [11-17], the peak was unequivocally identified as emodin.

Peak 7 was eluted at retention time of 73.8 min with maximal UV absorption at 223 and 286 nm. Deprotonated molecular ion at *m*/*z* 283 [M-H]^-^ was observed in its MS spectrum in the negative ion mode, which further generated a predominant ion at *m*/*z* 255 in MS^2^ spectrum owing to the loss of a neutral CO molecule. Other fragment ions at *m*/*z* 240 and 212 were also observed owing to the successive losses of a methyl unit and a CO molecule from 255. In positive ion mode, protonated molecular ion at *m*/*z* 285 [M + H]^+^ and sodium adduct ion at *m*/*z* 307 [M + Na]^+^ were observed in MS spectrum of the peak. Based on the MS data reported in publications [12-17] and the comparison with the standard, it was unequivocally identified as physcion.

## Method validation

### Calibration curves, LODs and LOQs

The calibration curve of each compound was performed with six appropriate concentrations in duplicate and constructed by plotting the peak areas versus the concentrations. As shown in Table 3, all calibration curves showed good linear regression (*R*^*2*^ > 0.9990) in a relatively wide range. The stock solution of each reference compound was further diluted to a series of concentrations with 75% methanol for LOD and LOQ. LODs for THSG, emodin and physcion were 98.08 ng/mL, 44.26 ng/mL and 56.77 ng/mL, respectively, and LOQs for THSG, emodin and physcion were 404.03 ng/mL, 180.56 ng/mL and 132.20 ng/mL, respectively, with injection volume of 10 *μ*L.

**Table 3:** **Regression data**, **LODs and LOQs for the three analytes tested in HPLC**-**UV chromatograms**

**Analyte**	**Calibration curve**^**a**^	***R***^**2**^	**Linear range (mg/L)**	**LOD**^**b **^**(ng/mL)**	**LOQ**^**c **^**(ng/mL)**
1	*y* = 29885 *x* - 210960	0.9990	0.404-252.5	98.08	404.03
2	*y* = 38332 *x* + 23549	0.9999	0.0443-138.325	44.26	180.56
3	*y* = 22565 *x* - 1352.2	0.9997	0.123-15.4	56.77	132.20

1: THSG.

2: emodin.

3: physcion.

^a^*y* is the peak area in UV chromatograms, *x* is the concentration (mg/L) of the analyte.

^b^ LOD refers to the limit of detection, s/n = 3.

^c^ LOQ refers to the limit of quantification, s/n = 10.

### Precision, repeatability and stability

The RSDs of the retention times and peak areas of the three analytes were taken as the measurements of precision and stability. The RSDs of the retention times and contents of the three analytes in dried samples (mg/g) were taken as the measurements of repeatability. As shown in Table 4, the overall RSD values were less than 3.00%, indicating that the developed method was satisfactory on quantification of THSG, emodin and physcion in PMC samples.

**Table 4:** Results of precision, repeatability and stability of the three analytes, expressed as RSD (%)

**Analyte**	**Precision**				**Repeatability (n = 5)**		**Stability (n = 6)**	
	**Intra**-**day RSD (%) (n = 5)**		**Inter**-**day RSD (%) (n = 9)**		**RSD (%)**		**RSD (%)**	
	**t**_**R**_^**a**^	**PA**^**b**^	**t**_**R**_	**PA**	**t**_**R**_	**Contents (%)**	**t**_**R**_	**PA**
1	0.58	1.24	2.29	1.98	0.78	1.25	1.69	2.85
2	0.62	0.88	1.98	2.01	0.51	1.69	0.96	2.31
3	0.49	1.58	1.86	2.89	0.66	2.08	1.33	2.44

^a^ Refers to retention time.

^b^ Refers to peak area.

### Quantification of THSG, emodin and physcion in PMC samples

The established HPLC-UV quantitative analytical method was successfully applied for simultaneous quantification on the three compounds in nineteen PMC samples (eight were from mainland China, and eleven were from local pharmacies of Hong Kong). The contents (mg/g dry weight) were calculated and summarized (n = 2) in Table 1.

Firstly, the results showed that the contents of each compound in different PMC samples varied markedly. To our surprise, the contents of THSG, emodin and physcion ranged from 0.268 to 41.361 mg/g, 0.010 to 1.295 mg/g and 0.013 to 1.394 mg/g, respectively. In addition, THSG was not detected (below LOD) in L-PMC-07, L-PMC-09 and L-PMC-10, emodin was not detected in L-PMC-07, L-PMC-08, L-PMC-09 and L-PMC-10, and physcion was not found in PMC-03, PMC-05, L-PMC-02, L-PMC-06, L-PMC-07, L-PMC-08, L-PMC-09 and L-PMC-10. The results indicated that significant differences of the concentrations of each compound in different PMC samples were found.

Secondly, according to the regulation of China pharmacopoeia (2010 edition) that the content of THSG in dried PMC sample should not be less than 0.20% (2.0 mg/g), only five samples in our study, including PMC-01, PMC-02, PMC-07, PMC-08 and L-PMC-11, were definitely qualified raw medicinal materials for clinic use. It was worth mentioning that one of the eleven local PMC samples from Hong Kong, L-PMC-11, had the highest content of THSG in all the tested samples, which was also the only one qualified sample from local pharmacy of Hong Kong. Emodin and physcion are not the specified chemical markers in China pharmacopoeia, but they usually exist in the plants from family Polygonaceae, which were also quantified in PMC or its related commercial product [18,19]. The data obtained in the present study showed that except the samples in which emodin were not detected, the content of emodin was the highest in PMC-02, however, the lowest, in L-PMC-02. In the same way, the content of physcion in PMC-02 was the highest and the one in L-PMC-05 was the lowest. The results also indicated that emodin and physcion were not the dominant chemical compounds in PMC compared with THSG.

Thirdly, the PMC samples tested in the present study were mainly from southlands of China. L-PMC-11 was found to have the highest content of THSG at 41.361 mg/g and relative higher contents of emodin (0.864 mg/g) and physcion (1.054 mg/g) in all the tested samples. But its origin was unknown. The sample from Yunnan, numbered PMC-02, had the highest contents of emodin (1.295 mg/g) and physcion (1.394 mg/g) as well as the second highest content of THSG (23.766 mg/g) in all the samples. However, the samples, in which the three analytes were not detected, were from different origins. Seeing from the results, we find that the qualities of PMC samples collected for the present study do not have necessary relations with their origins.

**Figure 4:**
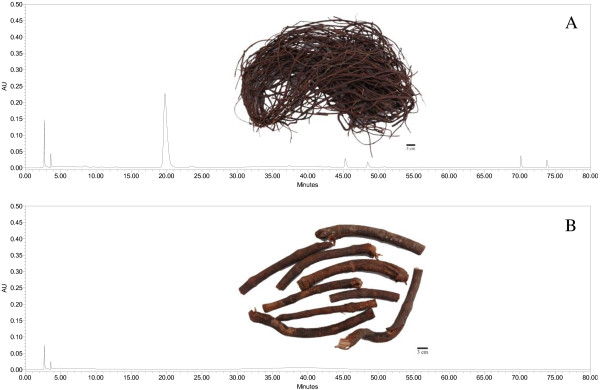
**Chromatographic fingerprints of L-PMC-11 (A) and L-PMC-10 (B) detected at 290 nm.** The embedded photos are original raw plant materials of L-PMC-11 and L-PMC-10, respectively. The figures show the original morphologies and the different fingerprints of the PMC samples.

The last but the most important, PMC is the dried caulis of *Polygonum multiflorum* Thunb., which is a perennial plant from family Polygonaceae. One of the descriptions of PMC in China pharmacopeia (2010 edition) is that their diameters are between 4 and 7 mm, but the diameters of PMC on market have great differences. The information about diameters of PMC samples in our study are summarized in Table 1. The contents results indicated that the diameters of the five qualified PMC samples, including PMC-01, PMC-02, PMC-07, PMC-08 and L-PMC-11, basically fell in the defined range (each sample had a few stems of which the diameters were out of the range) (Figure 4A). They also had higher contents of emodin and physcion than others. The diameters of L-PMC-07, L-PMC-09 and L-PMC-10, in which all the three analytes were undetectable, all exceeded the defined range. To our surprise, no obvious peak was detected in these samples (Figure 4B). As for PMC-05, PMC-06, L-PMC-04 and L-PMC-08, just a few branches of each sample were thin but most of them were thick, resulting in their unqualified qualities. Neither the sample (PMC-04) with diameter value lower than 4 mm nor the samples with diameter values more than 7 mm (PMC-03, L-PMC-01, L-PMC-02, L-PMC-03, L-PMC-05 and L-PMC-06) were unqualified. But it was worth noting that PMC-04 with the lowest diameter value had relative higher content of THSG in all the unqualified samples. So, we speculated that the content of THSG would increase with its growth. When the diameter values of its stems reached the range between 4 and 7 mm, its content of THSG would met with CP’s requirement and then it was the best harvesting time. In addition, as shown in Table 1, eight out of nineteen in our tested PMC samples were medicinal slices. All of them are unqualified ones according to the specification in CP (2010 edition), but we don’t think their qualities have positive correlations with processing methods since raw materials of PMC are just cut and dried in shade places to get medicinal slices generally. We speculate that it is still the diameters of stems’ of the raw materials that influence their qualities.

### Fingerprinting and principal component analyses

Although the quantification results can confirm the contents of THSG, emodin and physcion in a PMC sample, there is no way to know intuitively how similar a PMC sample to another one on the whole just by quantification a few compounds. Fingerprinting and chemometrics analyses, however, can show the chemical similarities between one and another one holistically and visually. Principal component analysis, one of the chemometrics, is an unsupervised mathematical procedure that transforms a number of possibly correlated variables into a smaller number of uncorrelated variables called principal components. Its operation can be thought of as revealing the internal structure of the data in a way which best explains the variance.

**Figure 5:**
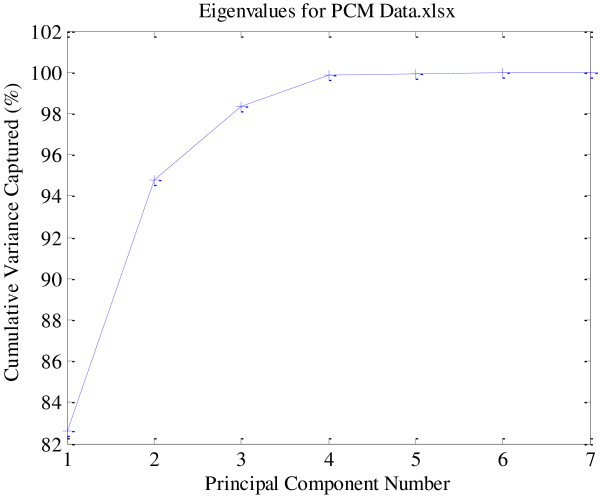
**The cumulative variance of the seven generated principal components.** The first two components account for 94.75% of total variance.

**Figure 6:**
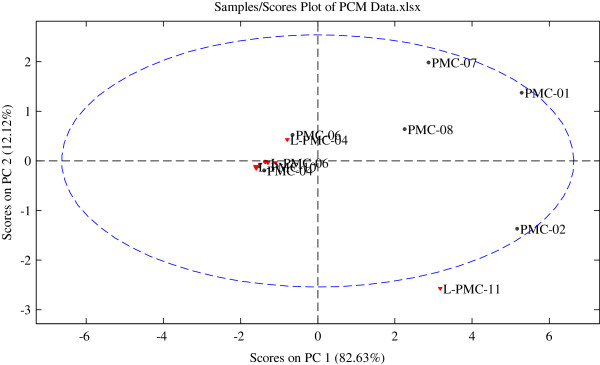
PCA scores plot of PMC samples.

Seven peaks marked 1 to 7 were selected as characteristic peaks in chromatographic fingerprints in the present study (Figure 1). Although peak 2 ~ peak 5 were tentative identified as emodin-8-O-*β*-D-glucoside, emodin-8-O-(6′-O-malonyl)-*β*-D-glucoside, physcion-8-O-*β*-D-glucoside and physcion-8-O-(6′-acetyl)-*β*-D-glucoside respectively based on the data in Table 2 and published literatures, due to the unavailability of reference compounds, they were not quantified. So, the variables of each sample consisted of contents of peak 1, peak 6 and peak 7 and PA/W (peak area divided by sample weight) values of peak 2 ~ peak 5. The data were exported to Excel (Microsoft, Inc., Belleview, WA) to form a two-dimensional matrix (nineteen samples versus seven variables) which was then exported to SOLO for PCA. A two-component (the first two components) model cumulatively accounted for 94.75% of total variance (Figure 5), based on which PCA scores plot (Figure 6) was generated. From the scores plot, we can see intuitively a very interesting phenomenon that PMC-01, PMC-02, PMC-07, PMC-08 and L-PMC-11, which were regarded as the only five qualified samples in the present study, are separated very well with other samples in PC1. They all get PC1 scores above zero, however, others obtain PC1 scores below zero. What’s more, L-PMC-11 is located outside the ellipse (95% confidence interval) because of its the highest content of THSG.

**Figure 7:**
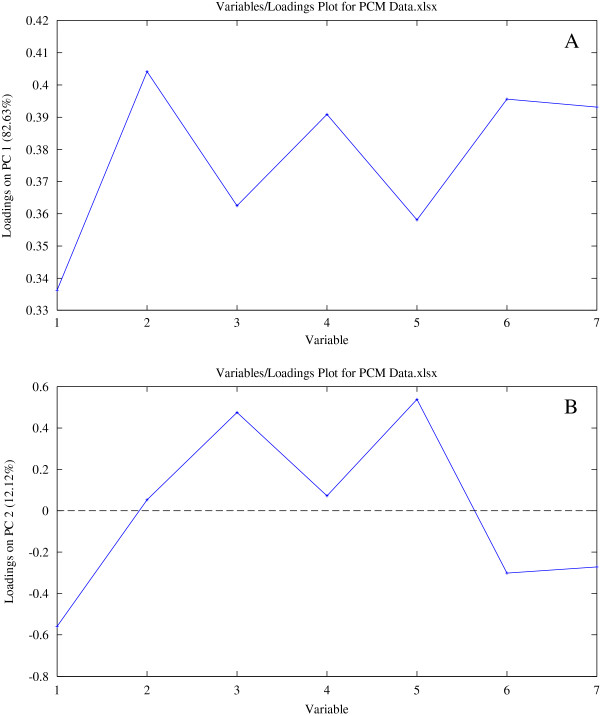
**PC1 (A) and PC2 (B) loadings plots of the seven variables.**

To find out variables contributing to the significant differences between different PMC samples, PC1 and PC2 loadings plots were generated. PC1 loadings plot (Figure 7A) indicates peak 2, peak 4, peak 6 and peak 7 are mainly responsible for the separation of samples on PC1 (*P* < 0.05). What it means is that higher PA/W values of peak 2 and peak 4 and higher contents of peak 6 and peak 7 will give higher PC1 scores, moving the positions of the samples to the right on PCA scores plot. PMC-01 gets the highest PA/W value of peak 2 in all the samples. PMC-02 gets the highest PA/W value of peak 4 and the highest contents of peak 6 and peak 7 in all the samples. So, the two samples are placed in the rightmost positions in scores plot. In the same way, L-PMC-11, PMC-07 and PMC-08 have higher contents of peak 6 and peak 7 or higher PA/W values of peak 2 and peak 4, making them get higher PC1 scores than others except PMC-01 and PMC-02.

According to PC2 loadings plot, peak 3 and peak 5 mainly contribute to high PC2 scores, however, peak 1, peak 6 and peak 7 contribute to low PC2 scores (*P* < 0.05). PMC-07 has the highest PA/W value of peak 3 and the second highest PA/W value of peak 5. PMC-01 has the highest PA/W value of peak 5 and the second highest PA/W value of peak 3. So, they are positioned on the top in scores plot, having higher PC2 scores. The highest content of peak 1 was found in L-PMC-11, and then in PMC-02. The highest PA/W value of peak 6 and the highest content of peak 7 were both obtained in PMC-02, and then in L-PMC-11. The above two reasons lead to the positions of the two samples at the bottom.

Other samples are clustered tightly around the corner due to their similar and low contents or PA/W values of the seven peaks.

All in all, scores plot shows the distributions of the tested samples intuitively and clearly, meanwhile, loadings plots indicate the influences of the variables on the positions of PMC samples. From the scores obtained in the present study, qualified and unqualified PMC samples can be distinguished easily and efficiently.

## Conclusions

For the first time, systematic HPLC/UV/MS chromatographic fingerprinting and quantitative analytical methods combined with principal component analysis were developed to analyze different PMC samples. The contents of THSG were found to have surprising relevance with the samples’ diameters. Diameters of the five qualified PMC samples basically fell in the specified range, which also had higher contents of emodin and physcion than others. However, diameters of the unqualified PMC samples generally exceeded the specified range. Seven characteristic peaks in chromatographic fingerprints marked 1 to 7 were identified, and based on the contents or PA/W values of the seven variables, PCA scores plot was generated. The finding in the present study provides a scientific basis for collecting PMC in the best time, and with the aid of PCA, unqualified PMC samples can be singled out from qualified ones easily and efficiently.

## Competing interests

The authors declare that they have no competing interests.

## Authors’ contributions

YSC and YLH initiated and design the study. The extraction and method developments were conducted by YZ and CPK. YZ drafted the manuscript. All authors contributed to data analyses and finalized the manuscript. All authors have read and approved the final version.

## Supplementary Material

Additional file 1: Figure S1 A Chromatograms of Polygoni Multiflori Caulis extracted with different solvents. **Figure S1 B.** The peak areas of THSG in different chromatograms of Polygoni Multiflori Caulis extracted with different solvents. **Figure S1 C.** The chromatograms of Polygoni Multiflori Caulis extracted with 75% methanol for three times. Click here for file
