# Supplementary material for: Quality assessment on Polygoni Multiflori Caulis using HPLC/UV/MS combined with principle component analysis
Source: Chem Cent J. 2013 Jun 24;7:106. doi: 10.1186/1752-153X-7-106 (PMC3695832; doi:10.1186/1752-153X-7-106)
Supplement: Additional file 1: Figure S1 A — Chromatograms of Polygoni Multiflori Caulis extracted with different solvents. Figure S1 B. The peak areas of THSG in different chromatograms of Polygoni Multiflori Caulis extracted with different solvents. Figure S1 C. The chromatograms of Polygoni Multiflori Caulis extracted with 75% methanol for three times. [file 1752-153X-7-106-S1.docx]

Extracted with Ethanol

2,3,5,4'-THT-2-O-*β*-D-glucoside

Extracted with 50% Methanol

2,3,5,4'-THT-2-O-*β*-D-glucoside

Extracted with 75% Methanol

2,3,5,4'-THT-2-O-*β*-D-glucoside

Extracted with Methanol

2,3,5,4'-THT-2-O-*β*-D-glucoside

Fig. A Chromatograms of Polygoni Multiflori Caulis extracted with different solvents

Fig. B The peak areas of 2,3,5,4'-THT-2-O-*β*-D-glucoside in different chromatograms of Polygoni Multiflori Caulis extracted with different solvents

2,3,5,4'-THT-2-O-*β*-D-glucoside

Extracted for the first time

2,3,5,4'-THT-2-O-*β*-D-glucoside

Extracted for the second time

2,3,5,4'-THT-2-O-*β*-D-glucoside

Extracted for the third time

Fig. C. The chromatograms of Polygoni Multiflori Caulis extracted with 75% methanol for three times.
